# Visual and Auditory Components in the Perception of Asynchronous Audiovisual Speech

**DOI:** 10.1177/2041669515615735

**Published:** 2015-11-30

**Authors:** Miguel A. García-Pérez, Rocío Alcalá-Quintana

**Affiliations:** Departamento de Metodología, Facultad de Psicología, Universidad Complutense, Madrid, Spain

**Keywords:** Synchrony judgments, perceived onset, audiovisual speech, multisensory integration

## Abstract

Research on asynchronous audiovisual speech perception manipulates experimental conditions to observe their effects on synchrony judgments. Probabilistic models establish a link between the sensory and decisional processes underlying such judgments and the observed data, via interpretable parameters that allow testing hypotheses and making inferences about how experimental manipulations affect such processes. Two models of this type have recently been proposed, one based on independent channels and the other using a Bayesian approach. Both models are fitted here to a common data set, with a subsequent analysis of the interpretation they provide about how experimental manipulations affected the processes underlying perceived synchrony. The data consist of synchrony judgments as a function of audiovisual offset in a speech stimulus, under four within-subjects manipulations of the quality of the visual component. The Bayesian model could not accommodate asymmetric data, was rejected by goodness-of-fit statistics for 8/16 observers, and was found to be nonidentifiable, which renders uninterpretable parameter estimates. The independent-channels model captured asymmetric data, was rejected for only 1/16 observers, and identified how sensory and decisional processes mediating asynchronous audiovisual speech perception are affected by manipulations that only alter the quality of the visual component of the speech signal.

## Introduction

How humans perceive asynchrony in audiovisual speech is an active area of research. Because asynchrony is only momentarily detected in continuous speech streams and observers cannot provide unequivocal information about the articulations that caused it, perception of asynchronous speech is often investigated using single-syllable utterances. Data are collected with a temporal-order judgment (TOJ) task or a binary synchrony judgment (SJ2) task. In both cases, auditory and visual components of a speech signal are decoupled and presented with asynchronies that vary across trials. In TOJ tasks, observers indicate whether the audio or the video component was perceptually ahead; in SJ2 tasks, observers indicate whether audio and video were perceptually synchronous. A psychometric function is then fitted to the proportion of video-first (VF) judgments (TOJ tasks) or SJ2 tasks as a function of audiovisual offset, usually with the only goal of estimating the point of subjective simultaneity (PSS) or the difference limen (DL). Thus, cumulative Gaussians or logistic functions are fitted to TOJ data and scaled Gaussians are fitted to SJ2 data. Although the functions fitted to TOJ data can be referred to an observer model, this is not true for the functions fitted to SJ2 data. We will argue that the implied model for TOJ data fails to represent all the relevant processes, and that the psychometric function fails to capture some common characteristics of empirical data. Sometimes functions are fitted piecewise within separate ranges of audiovisual offsets (Kohlrausch, van Eijk, Juola, Brandt, & van de Par, 2013; van Eijk, Kohlrausch, Juola, & van de Par, 2008, 2010), which better accommodates the path of SJ2 data but cannot be referred to any observer model either.

Although fitting such psychometric functions is justifiable, some characteristics of data suggest that the functions are inadequate. The sigmoidal shape of TOJ data is usually asymmetric and shows an intermediate region of reduced slope that cannot be captured by cumulative Gaussian or logistic functions (see Diederich & Colonius, 2015; García-Pérez & Alcalá-Quintana, 2015a, 2015b). Also, the inverted-U shape of SJ2 data usually shows a broad plateau and different drop-off rates on either side, features that cannot be captured by symmetric and sharply peaked Gaussians. More important, the (only two) parameters of these functions cannot be linked to the various processes determining observers’ responses. Thus, the effects of manipulations aimed at affecting such processes can only be assessed at an undifferentiated level (or via PSSs and DLs) with no means to identify which processes were affected and how.

Probabilistic models of the processes underlying timing judgments yield psychometric functions that capture the afore-mentioned features with interpretable parameters. Such models represent sensory aspects (i.e., the distribution of the perceived onset of each of the signals involved) and decisional aspects pertaining to how observers use sensory information to give a response. The explicit representation of such processes and the functional characterization of their operation through model-based psychometric functions allow assessing the effects of experimental manipulations and testing hypotheses about which of the underlying processes are affected. For instance, García-Pérez and Alcalá-Quintana (2012a) used an independent-channels (IC) model to show that response errors that are sometimes clearly evident in the data can explain the absence of theoretical properties that once were deemed inherent to timing judgments. Also, García-Pérez and Alcalá-Quintana (2012b, 2015a, 2015b) used the same model to show that observed differences in performance across timing judgment tasks can be explained by differences in decisional aspects, with the sensory component remaining invariant when conditions are identical across tasks. Other studies confirmed the utility of the IC model to interpret the operation of underlying timing processes (see e.g., Matsuzaki et al., 2014; Regener, Love, Petrini, & Pollick, 2014).

Magnotti, Ma, and Beauchamp (2013) recently proposed another model based on different principles—which they called causal inference of multisensory speech (CIMS) model—and fitted it across the four conditions in their empirical study. The IC and CIMS models account for observed performance on different assumptions about the underlying processes. The purpose of this article is to compare the structural and functional characteristics of the two models, also assessing their capability to fit this data set and the interpretations they provide about observed differences in performance across conditions.

Although both models have been fully described elsewhere, the next section describes them in some detail with an eye to stressing their similarities and differences. The SJ2 data of Magnotti et al. (2013) and our fitting approach are subsequently described, and the results of the IC fit are presented and discussed in comparison with those of the CIMS model. The section Discussion presents further comparisons of the models and reflections on methodological and experimental practices that provide better grounds for interpretation of the effects of experimental manipulations.

## Models

### IC Model

In the IC model, visual and auditory signals from the speech stimulus are independently processed through their sensory pathways to render perceived times of occurrence (onsets) and a timing judgment results upon application of a decision rule to the perceived onsets in a trial. A full presentation of the model can be found in García-Pérez and Alcalá-Quintana (2012b) but it is briefly presented next in terms adapted to audiovisual speech stimuli.

**Figure 1.**
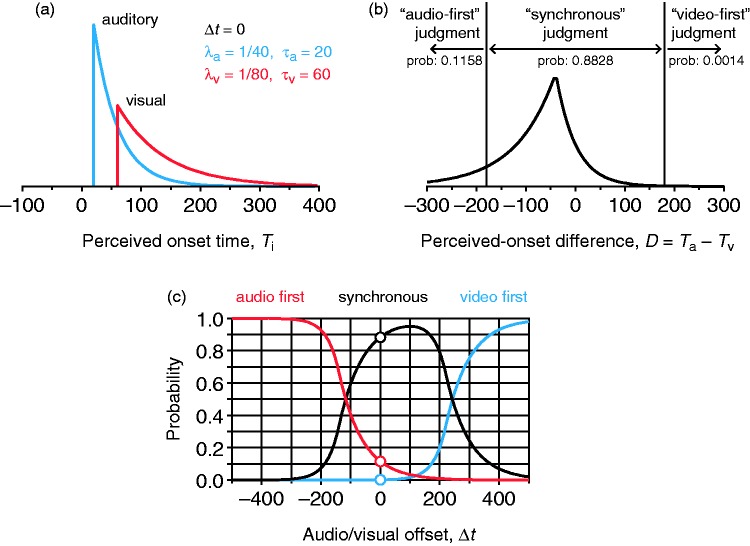
Independent-channels model of timing judgments in audiovisual speech. (a) Shifted exponential distributions of perceived visual onset (red curve) of the visual component assumed to occur physically at time 0 and perceived auditory onset (blue curve) of the auditory component occurring simultaneously (i.e., at an audiovisual offset Δ*t* = 0). Parameters of each distribution as indicated in the inset. (b) Bilateral exponential distribution of perceived-onset differences (curve) and boundaries in the decision space (vertical lines at *D* = ± δ with δ = 180), determining the probability of each type of judgment. (c) Psychometric function for each type of judgment as a function of audiovisual offset Δ*t*. Circles denote the probabilities indicated in (b) for Δ*t* = 0.

The *perceived onset times T*_v_ and *T*_a_ of the visual and auditory components of an audiovisual speech signal are random variables with densities *g*_v_ and *g*_a_ given by the shifted exponential distributions

(1) $$g_{i}(t)=\lambda_{i}\exp[-\lambda_{i}(t-(\Delta t_{i}{+\tau }_{i}))],\;t\geq \Delta t_{i}{+\tau }_{i},i\in \{\text{v},\text{a}\}$$

where Δ*t_i_* is the onset of component *i*, λ*_i_* is the exponential rate parameter, and τ*_i_* is a processing delay. Exponential distributions capture the causality that governs perceived onsets: The onset of a stimulus cannot be perceived before the stimulus has been presented. Although other distributions also capture this characteristic, Gaussian distributions certainly do not. The origin of time is at the onset of the visual component so that Δ*t*_v_ = 0 and Δ*t* ≡ Δ*t*_a_ is the asynchrony created by manipulating the audiovisual offset, where Δ*t* < 0 (Δ*t* > 0) reflects that the auditory component precedes (lags) the visual component. Figure 1(a) shows distributions when Δ*t* = 0 (i.e., no artificial audiovisual offset); the mean and variance are μ*_i_* = 1/λ*_i_* + τ*_i_* + Δ*t_i_* and $\sigma_{i}^{2}$ = ${1/\lambda }_{i}^{2}$, respectively. These distributions reflect the differential accuracy with which visual and auditory onsets can be perceived.

Observer’s judgments arise from a decision rule applied to the perceived-onset difference *D* = *T*_a_ − *T*_v_, which has the bilateral exponential distribution

(2) $$f(d;\Delta t)=\{\frac{\lambda_{\text{a}}\lambda_{\text{v}}}{\lambda_{\text{a}}+\lambda_{\text{v}}}\exp[\lambda_{\text{v}}(d-\Delta t-\tau )]\text{if}d\leq \Delta t+\tau \frac{\lambda_{\text{a}}\lambda_{\text{v}}}{\lambda_{\text{a}}+\lambda_{\text{v}}}\exp[-\lambda_{\text{a}}(d-\Delta t-\tau )]\text{if}d>\Delta t+\tau$$

where τ = τ_a_ − τ_v_ is the *auditory advantage* reflecting how much earlier (if τ < 0) or later (if τ > 0) the shortest possible perceived auditory onset may occur compared with the shortest possible perceived visual onset in a synchronous stimulus (see Figure 1(a)). Figure 1(b) shows the distribution of perceived-onset differences for the case in Figure 1(a). For arbitrary Δ*t*, the mean perceived-onset difference is μ*_d_* = μ_a_ − μ_v_ = 1/λ_a_ − 1/λ_v_ + τ + Δ*t* and the variance is $\sigma_{d}^{2}$ = ${1/\lambda }_{\text{a}}^{2}$ + ${1/\lambda }_{\text{v}}^{2}$.

A *resolution* parameter δ (see Figure 1(b)) limits the ability of the observer to tell small differences in perceived onset. Thus, audio-first (AF) judgments occur when *D* is sufficiently large and negative (*D* < −δ), VF judgments occur when *D* is sufficiently large and positive (*D* > δ), and synchronous (S) judgments occur when *D* is below the resolution limit (−δ ≤ *D* ≤ δ). The probability of each judgment varies with Δ*t*, as Δ*t* shifts the distribution of *D*. Figure 1(c) shows psychometric functions describing how these probabilities vary with Δ*t*. They are given by

(3a) $$\Psi_{\text{AF}}(\Delta t)=\int_{-\infty }^{-\delta }f(z;\Delta t)\text{d}z=F(-\delta ;\Delta t)$$

(3b) $$\Psi_{\text{S}}(\Delta t)=\int_{-\delta }^{\delta }f(z;\Delta t)\text{d}z=F(\delta ;\Delta t)-F(-\delta ;\Delta t)$$

(3c) $$\Psi_{\text{VF}}(\Delta t)=\int_{\delta }^{\infty }f(z;\Delta t)\text{d}z=1-F(\delta ;\Delta t)$$

where

(4) $$F(d;\Delta t)=\int_{-\infty }^{d}f(z;\Delta t)\text{d}z=\{\frac{\lambda_{\text{a}}}{\lambda_{\text{a}}+\lambda_{\text{v}}}\exp[\lambda_{\text{v}}(d-\Delta t-\tau )]\text{if}d\leq \Delta t+\tau 1-\frac{\lambda_{\text{v}}}{\lambda_{\text{a}}+\lambda_{\text{v}}}\exp[-\lambda_{\text{a}}(d-\Delta t-\tau )]\text{if}d>\Delta t+\tau$$

In SJ2 tasks, AF and VF judgments are aggregated into asynchronous (A) judgments and only the psychometric function in equation 3(b) is observed. The IC model can be extended to cover response errors (García-Pérez & Alcalá-Quintana, 2012a, 2012b) but the extension is not used here because the data to be presented below did not show evidence of errors (see section Discussion). The IC model thus describes performance in SJ2 tasks with four parameters: λ_a_, λ_v_, τ, and δ.

Three characteristics of Ψ_S_ (black curve in Figure 1(c)) under the IC model match empirical characteristics of SJ2 data. First, Ψ_S_ is asymmetric when λ_a_ ≠ λ_v_, being skewed in one direction or the other according to the sign of λ_a_ − λ_v_. Second, Ψ_S_ peaks at $\Delta t_{\text{peak}}=\delta \frac{\lambda_{\text{a}}-\lambda_{\text{v}}}{\lambda_{\text{a}}+\lambda_{\text{v}}}-\tau$, reflecting the empirical fact that the PSS is usually away from Δ*t* = 0. Third, Ψ_S_ has a plateau whose breadth depends on the width of the interval [−δ, δ].

### CIMS Model

The CIMS model (Magnotti et al., 2013) uses a Bayesian framework that can be ultimately referred to a decision space analogous to that in our Figure 1(b). The CIMS model assumes a distribution of “measured asynchronies” (analogous to the perceived-onset differences in our Figure 1(b)) that is instead normal with variance σ^2^ and mean equal to the audiovisual offset Δ*t* in the current trial. Measured asynchronies are differences between independent and normally distributed perceived auditory and visual onsets, but the parameters of these distributions are not explicitly included in the model so that the two components of σ^2^ (one from each stimulus) cannot be separated. The consequence of a normal distribution of measured asynchronies is that the psychometric function Ψ_S_ arising from the CIMS model must be symmetric. The model also assumes a decision space with three regions as in our Figure 1(b) so that the resultant Ψ_S_ can have a plateau, but these boundaries are not determined by an independent resolution parameter. Instead, they are placed according to a Bayesian hypothesis-testing approach. Specifically, the CIMS model considers the observer’s decision as an optimal test of the hypothesis that the visual and auditory components of the stimulus have a single cause (a condition denoted *C* = 1) against the hypothesis that they have two causes (a condition denoted *C* = 2). This implies a likelihood-ratio analysis of the observer’s prior distributions of measured asynchronies when *C* = 1 (assumed normal with mean μ_1_ and variance $\sigma^{2}+\sigma_{1}^{2}$) and when *C* = 2 (also assumed normal with mean μ_2_ and variance $\sigma^{2}+\sigma_{2}^{2}$), taking into account the observer’s bias toward assuming a single cause (given by the additional parameter *p_C_*_ = 1_). Magnotti et al. (2013) set μ_1_ = 0 for an arbitrary and inconsequential anchor. The optimal decision rule states that synchrony is to be reported when the likelihood of *C* = 1 exceeds the likelihood of *C* = 2, yielding boundaries at

(5) $$\delta =-\mu_{2}\frac{\sigma^{2}+\sigma_{1}^{2}}{\sigma_{2}^{2}-\sigma_{1}^{2}}\pm \sqrt{\frac{\left(\sigma^{2}+\sigma_{1}^{2})(\sigma^{2}+\sigma_{2}^{2}\right)}{\sigma_{2}^{2}-\sigma_{1}^{2}}(\frac{\mu_{2}^{2}}{\sigma_{2}^{2}-\sigma_{1}^{2}}+2\log(\frac{p_{C=1}\sqrt{\sigma^{2}+\sigma_{2}^{2}}}{(1-p_{C=1})\sqrt{\sigma^{2}+\sigma_{1}^{2}}}))}$$

The model has five free parameters (μ_2_, σ, σ_1_, σ_2_, and *p_C_*_ = 1_) and the resultant psychometric function is

(6) $$\Psi_{\text{S}}(\Delta t)=\Phi (\frac{\delta^{+}-\Delta t}{\sigma })-\Phi (\frac{\delta^{-}-\Delta t}{\sigma })$$

where Φ is the unit-normal cumulative distribution function, and δ^−^ and δ^+^ are the boundaries written out in compact form in equation (5). Note that the boundaries defined in equation (5) are monotonic in σ when μ_2_, σ_1_, σ_2_, and *p_C_*_ = 1_ have fixed values. Hence, δ^−^ and δ^+^ covary with the standard deviation σ, the midpoint (δ^− ^+ δ^+^)/2 is an increasing (decreasing) function of σ when μ_2_ < 0 (μ_2_ > 0), and the width δ^+ ^− δ^−^ is an increasing function of σ.

### Structural and Functional Comparison of the IC and CIMS Models

**Figure 2.**
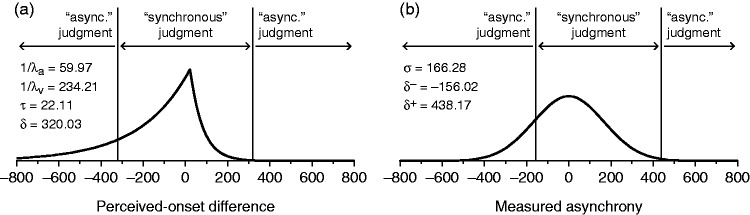
Structural comparison of the independent-channels and causal inference of multisensory speech models. (a) Distribution of perceived-onset differences when Δ*t* = 0 (curve) and decision boundaries (vertical lines) in the IC model. Parameter values come from fitting the IC model to data from observer #4 in condition 4 (see section Results). (b) Distribution of measured asynchronies when Δ*t* = 0 (curve) and decision boundaries (vertical lines) in the CIMS model. Parameter values come from fitting the CIMS model to the same data. For the resultant psychometric functions under each model, see the rightmost panel in the second row of Figures 3 and 4 below.

Figure 2 shows a graphical comparison of the IC and CIMS models using estimated parameter values for an actual observer. The IC model (Figure 2(a)) assumes a bilateral exponential distribution of perceived-onset differences with mean μ*_d_* = 1/λ_a_ − 1/λ_v_ + τ + Δ*t* and variance $\sigma_{d}^{2}$ = ${1/\lambda }_{\text{a}}^{2}$ + ${1/\lambda }_{\text{v}}^{2}$ and partitions the continuum symmetrically into three regions with boundaries at ± δ. In contrast, the CIMS model (Figure 2(b)) assumes a normal distribution of measured asynchronies with mean Δ*t* and variance σ^2^ and partitions the continuum also into three regions but asymmetrically with boundaries at δ^−^ and δ^+^ determined by σ^2^ and other model parameters. (For a summary list of parameters in each model, see Table 1 in the Supplementary Information.)

Besides the different sensory and decisional aspects represented by the parameters of each model and their different distributional assumptions, another conspicuous difference is that the IC model involves a centered partition and a distribution of perceived-onset differences whose mean is displaced from Δ*t*, whereas the CIMS model involves a displaced partition and a distribution of measured asynchronies centered on Δ*t*. This difference is inconsequential as far as the resultant psychometric functions are concerned: Rigid shifts of the distribution and the boundaries do not affect the area within the central region, which is what determines the shape of the psychometric function. Nevertheless, the difference is relevant upon inferring whether a shift of the observed psychometric function with respect to Δ*t* = 0 is due to perceptual processes or to decisional biases. This is a theoretical issue that cannot be solved with SJ2 data or, generally, with data from any variant of the method of single stimuli (see García-Pérez & Alcalá-Quintana, 2013; see also García-Pérez & Peli, 2014; Yarrow, Jahn, Durant, & Arnold, 2011). In any case, the IC model attributes shifts to perceptual processes, whereas the CIMS model attributes them to decisional bias.

A fundamental aspect on which the IC and CIMS models differ is the identifiability of their parameters. The identifiability of the IC model has been discussed and demonstrated elsewhere (see García-Pérez & Alcalá-Quintana, 2012a, 2012b). In contrast, the nonidentifiability of the CIMS model can easily be demonstrated. As seen in equation (6) and Figure 2(b), the psychometric function arising from the CIMS model depends only on three parameters (σ, δ^−^, and δ^+^) although the latter two are derived in turn from four additional parameters (μ_2_, σ_1_, σ_2_, and *p_C_*_ = 1_) besides σ itself (see equation (5)). Nonidentifiability can easily be appreciated by noting that, for fixed σ, there is an infinite set of values for μ_2_, σ_1_, σ_2_, and *p_C_*_ = 1_ that render the same values for δ^−^ and δ^+^ in equation (5) and, hence, the same psychometric function. The infinite set of solutions cannot be expressed analytically but alternative and very disparate sets can easily be obtained numerically. For an example involving the case shown in Figure 2(b), in which σ = 166.28 and (μ_2_, σ_1_, σ_2_, *p_C_*_ = 1_) = (−25.42, 73.10, 106.24, 0.52), it can be easily verified that the alternative sets (μ_2_, σ_1_, σ_2_, *p_C_*_ = 1_) = (−140.92, 133.60, 251.59, 0.48) or (μ_2_, σ_1_, σ_2_, *p_C_*_ = 1_) = (−0.03, 30.65, 30.75, 0.50) among many others also render (δ^−^, δ^+^) = (−156.02, 438.17) and, hence, the same psychometric function. (This result cannot be reproduced exactly from the rounded-off values printed here.) In practice, parameter-estimation algorithms return only one of the functionally equivalent solutions that are there to be found. Which one that is depends on the search method that is used and also on the starting values and the boundaries of the parameter space. The ultimate consequence of a multiplicity of solutions that account for the same data is that the (single) resultant set of estimated parameter values cannot be interpreted in terms of the underlying processes: A completely different interpretation would arise from any other of the solutions that might have been found. The nonidentifiability of the CIMS model has additional consequences in a joint fit across conditions, as discussed in the next section.

## Data and Model Fitting

Magnotti et al. (2013) collected data from 16 observers at each of 15 audiovisual offsets under four within-subjects conditions: two levels of degradation of the talking face image (sharp vs. blurred) factorially combined with two levels of visual intelligibility of the spoken word (high vs. low). They fitted the CIMS model jointly across conditions under the constraint that μ_2_, σ_1_, σ_2_, and *p_C_*_ = 1_ have common values in all conditions, whereas σ varies across conditions. Thus, eight parameters were estimated: four common parameters plus an additional parameter in each of the stimulus conditions. They made available computer code to replicate their fit, which we used to estimate the parameters whose analysis is presented in section Results. We also wrote our own code for reasons discussed next.

The nonidentifiability demonstrated in the preceding section revealed that only three parameters are involved in the CIMS model for an isolated condition: Parameters μ_2_, σ_1_, σ_2_, and *p_C_*_ = 1_ are combined with σ in a nonalgebraic form via equation (5) to produce the only two additional parameters δ^−^ and δ^+^. This reduction does not apply under the joint fit because the resultant δ^−^ and δ^+^ for each condition arise from a nonalgebraic combination of common and unique parameters. Thus, the actual number of functional parameters in the joint fit cannot be determined analytically. Because this number has implications when assessing goodness of fit, we investigated nonidentifiability and the possibility of parameter reduction numerically. For this purpose, we implemented the CIMS model into a multidimensional parameter-estimation method with the same routine used for the IC model (see below), which explores the parameter space simultaneously in all dimensions (compared with the sequential strategy of Magnotti et al., 2013), and we used it to fit the joint model under different conditions. When the parameter space was broad, the best-fitting solution was such that for almost all observers estimates of μ_2_ were negligibly different from zero, estimates of σ_1_ and σ_2_ were very close to one another, and estimates of *p_C_*_ = 1_ were negligibly different from 0.5. Compared with the results obtained with the code made available by Magnotti et al. (2013) to replicate their fit, estimates of μ_2_, σ_1_, σ_2_, and *p_C_*_ = 1_ were meaningfully different, although the fitted psychometric functions were indistinguishable by eye and the negative log-likelihoods were minimally smaller (by less than 1% on average). We reran our code using a narrow range for σ_1_ (80 units in breadth, randomly placed for each observer) and a range for σ_2_ such that σ_2_ > σ_1_ + 10. The results were analogous as regards quality of fit: Values for the negative log-likelihood were almost identical to those obtained in the preceding run and estimated psychometric functions were again indistinguishable. Yet, estimates of μ_2_, σ_1_, σ_2_, and *p_C_*_ = 1_ changed dramatically because now the algorithm returned the equivalent best-fitting solution within the narrow range defined for each observer. These results demonstrate the nonidentifiability of the CIMS model also under the joint fit and show that the multiple solutions that can be found are functionally equivalent. It also explains the different estimates returned by the code made available by Magnotti et al. (2013) upon reruns because each run uses a different random starting point and the parameter space is explored sequentially. This is also the reason that results to be presented in the next section for the CIMS model differ from those reported by Magnotti et al. (2013) for the same data, although parameter estimates were obtained with the same computer code in both cases.

To explore how many of the nominally four common parameters are identifiable in the joint fit, we conducted a thorough study in which the model was fitted in separate runs in which (a) each of the four individual parameters was given a fixed value one at a time and (b) each pair of parameters were given fixed values, also one pair at a time. None of the 10 cases produced a result that was functionally similar to that described in the preceding paragraph, and the fit was very poor in many cases. The implication is that the nonidentifiability of the CIMS model in the joint fit is irreducible: None of the four common parameters is disposable, surely because the only unique parameter per experimental condition (σ) cannot make up for the lack of flexibility that less than four common parameters permit. In sum, the joint fit of the CIMS model actually involves eight parameters despite its nonidentifiability and, with an exception to be discussed in section Results, this is the number of parameters that we will use in goodness-of-fit analyses. We did not consider the alternative route of using the number of functional parameters per condition (3, for a total of 12 across the four conditions) because it penalizes the CIMS model.

As for the IC model, we also fitted it jointly across conditions. Because experimental manipulations did not alter the auditory component of the stimulus, the distribution of perceived auditory onsets was assumed to be invariant and parameter λ_a_ was thus estimated to be common across conditions. Parameter τ_a_ must also be invariant across conditions although it is inextricably combined with parameter τ_v_ to yield the model parameter τ. Thus, the estimated τ must have a fixed component from the distribution of perceived auditory onsets and a condition-dependent component from the distribution of perceived visual onsets. Manipulations of the visual component of the stimulus must affect the distribution of perceived visual onsets because blurred images or low visual intelligibility surely hamper the identification of critical lip movements. Thus, parameter λ_v_ varied across conditions, as did parameter τ for the reason stated before. Finally, parameter δ was also allowed to vary across conditions for empirical and theoretical reasons discussed elsewhere (see García-Pérez & Alcalá-Quintana, 2012b, 2015a, 2015b). In sum, the joint fit of the IC model involved 13 parameters: a common parameter plus three additional parameters in each of the four stimulus conditions.

Model parameters were estimated by maximizing the joint log-likelihood equation

(7) $$L(R|\theta )=\sum_{j=1}^{J}\sum_{i=1}^{N}S_{i}^{\left(j\right)}\log(\Psi_{\text{S}}^{\left(j\right)}(\Delta t_{i}))+A_{i}^{\left(j\right)}\log(1-\Psi_{\text{S}}^{\left(j\right)}(\Delta t_{i}))$$

across the *J* = 4 conditions, where the parenthetical superscript *j* denotes the condition (1: sharp/high; 2: sharp/low; 3: blurred/high; 4: blurred/low), **R** is the set of responses across conditions, **θ** = ($\lambda_{\text{a}}$, $\lambda_{\text{v}}^{\left(1\right)}$, τ^(1)^, δ^(1)^, $\lambda_{\text{v}}^{\left(2\right)}$, τ^(2)^, δ^(2)^, $\lambda_{\text{v}}^{\left(3\right)}$, τ^(3)^, δ^(3)^, $\lambda_{\text{v}}^{\left(4\right)}$, τ^(4)^, δ^(4)^) is the vector of free parameters, {#x00394;*t*_1_, Δ*t*_2_,…, Δ*t_N_*} is the set of *N* = 15 audio or visual offsets at which data were collected, and $S_{i}^{\left(j\right)}$ and $A_{i}^{\left(j\right)}$ are the counts of observed synchronous and asynchronous responses at Δ*t_i_* in condition *j*. General-purpose software for fitting the IC model (Alcalá-Quintana & García-Pérez, 2013) was adapted and implemented in fortran to maximize equation (7) using the NAG subroutine e04jyf (Numerical Algorithms Group, 1999). The parameter space spanned the ranges [1/500, 1/35] for λ_a_ and λ_v_, the range [−300, 300] for τ, and the range [0, 600] for δ. Four initial values were defined for each parameter and combined to yield 4^4^ starting points. For each starting point, a vector of estimates and the corresponding likelihood-ratio goodness-of-fit statistic *G*^2^ were obtained; the final solution was the vector for which divergence was lowest. Use of a large number of starting points that cover the parameter space evenly guards against finding only a local maximum. Several simulation studies have shown that this strategy is efficacious (Alcalá-Quintana & García-Pérez, 2013; García-Pérez & Alcalá-Quintana, 2012a, 2012b).

## Results

### Psychometric Functions

**Figure 3.**
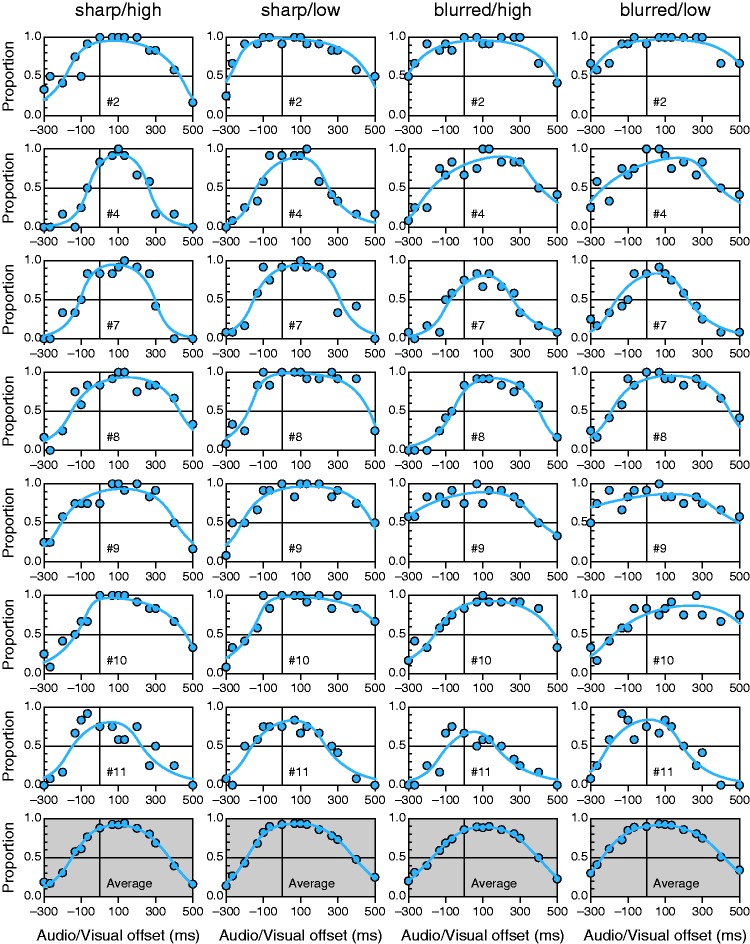
Data from Magnotti et al. (2013) and fitted independent-channels model psychometric functions. Each row pertains to the observer whose number is indicated in the inset (for the remaining observers, see the Supplementary Information); each column pertains to the condition indicated at the top. The bottom row displays average data across all 16 observers and the average of the corresponding fitted curves in each condition.

Figure 3 shows empirical data and fitted psychometric functions from the IC model in each condition for selected observers, also showing a summary row for average data and average fitted curves. Individual plots for the remaining observers are presented in the Supplementary Information. Table 2 (also in the Supplementary Information) lists parameter estimates as well as the value and *p* value of the *G*^2^ statistic for each observer.

The fit seems good: The *G*^2^ statistic was only marginally significant for observer #11 (seventh row in Figure 3). Lack of symmetry in the data is apparent in many panels, and the IC model accommodates well this characteristic. Visual inspection also reveals that the two visual intelligibility conditions (high vs. low) render similar results under each visual degradation condition (sharp vs. blurred), whereas larger differences can be seen across visual degradation conditions under either visual intelligibility condition. Specifically, the drop-off toward increasingly negative offsets is generally more abrupt with sharp images (first and second columns in Figure 3) than it is with blurred images (third and fourth columns in Figure 3), regardless of the visual intelligibility condition.

**Figure 4.**
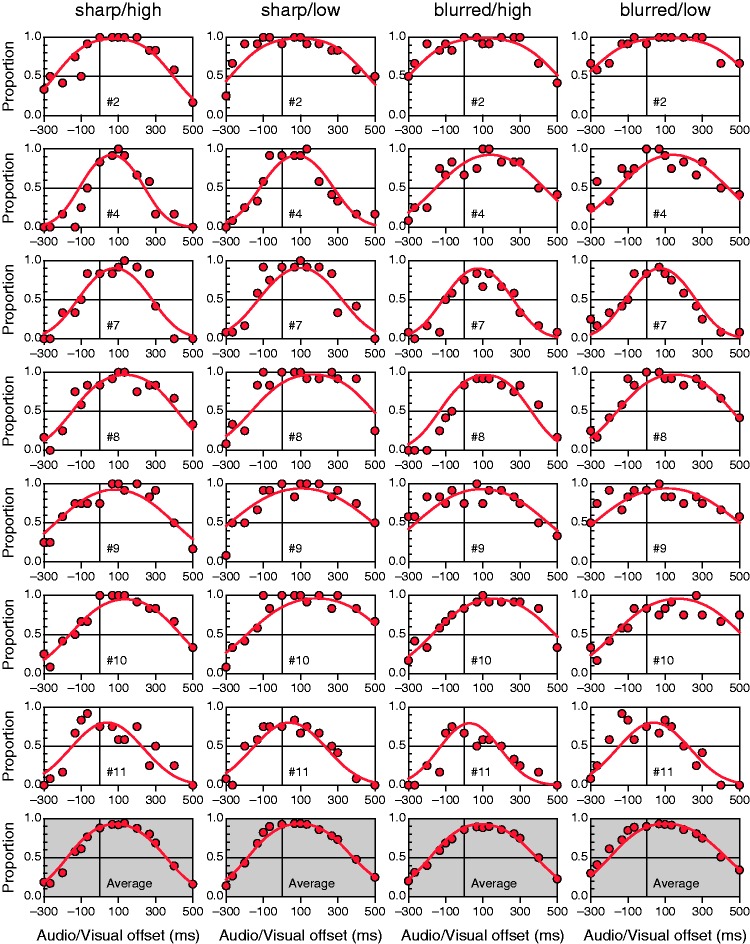
Data and fitted causal inference of multisensory speech model psychometric functions. Layout as in Figure 3. Estimated parameters were obtained with the code made available by Magnotti et al. (2013). Note that at the average level (bottom row), the CIMS model functions display systematic and patterned departures with respect to the data, which were not observed for the IC model (compare with the bottom row in Figure 3).

For comparison, Figure 4 shows fitted functions from the CIMS model for the same observers (for the remaining observers, see the Supplementary Information). At the 5% significance level, the *G*^2^ statistic rejected the CIMS model for eight of the 16 observers. Compared with Figure 3, the fit seems worse mainly because asymmetries cannot be captured by the symmetric functions imposed by the CIMS model. Thus, curves generally depart systematically from data in different directions at large positive and large negative audiovisual offsets. These systematic departures are more clearly visible for average data (compare the bottom rows in Figures 3 and 4).

We also compared model fits through the Bayesian Information Criterion (BIC), which takes into account the number of parameters in each model. The BIC was computed on a condition-by-condition basis identically for both models as −2logL + *k*log(*n*), where logL is the log-likelihood of the data given parameter estimates, *k* is the number of parameters involved in that condition, and *n* is the number of observations. Computing the BIC separately for each experimental condition requires also an adjustment of the number of parameters because only the parameters that apply in each condition must be considered. In the IC model, each condition implies four parameters (the three unique parameters for that condition plus the only common parameter) so *k* = 4. In the CIMS model, analogous considerations yield five parameters per condition (the four common parameters plus the only unique parameter for each condition), but using this number would be unfair on consideration that only three functional parameters exist per condition. Thus, we computed the BIC for the CIMS model in each condition using *k* = 3. It might be argued that common parameters should be evenly split across conditions for this analysis, which would render *k* = 3.25 for the IC model and *k* = 2 for the CIMS model. We repeated the analyses with these values but the results (not reported below) were virtually identical because the models still differ by about one parameter only.

**Figure 5.**
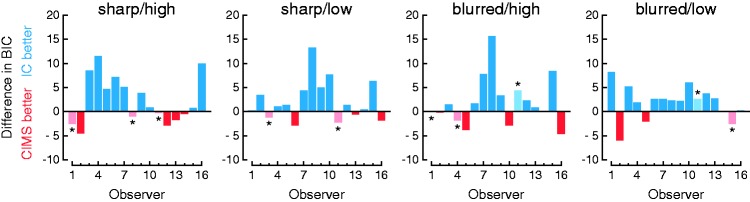
Statistical comparison of the independent-channels and causal inference of multisensory speech models. Each panel shows the difference in BIC for each observer in each condition. Positive values (blue bars) indicate a lower BIC for the IC model; negative values (red bars) indicate a lower BIC for the CIMS model. Stars and lighter bars indicate observers for whom the model with the lower BIC was nevertheless rejected by the *G*^2^ statistic, something that occurred very often for the CIMS model.

The results (see Figure 5) are somewhat mixed although they favor the IC model across the board, particularly when data show asymmetries. Consider the case of observer #8 (fourth row in Figures 3 and 4). The range of audiovisual offsets tested is sufficiently broad for this observer to show clear evidence of asymmetry in conditions 2 (sharp/low), 3 (blurred/high), and 4 (blurred/low) but only mild evidence in condition 1 (sharp/high). Naturally, the IC model outperforms the CIMS model by the BIC in conditions 2 to 4 and is only outperformed by the CIMS model in condition 1 (see Figure 5). Similarly, for observer #7 (third row in Figures 3 and 4) the IC model outperforms the CIMS model in all conditions for the same reason. In contrast, when the data do not show clear evidence of asymmetries (e.g., for observer #2 in conditions 1 and 4; first row in Figures 3 and 4), the extra (but unnecessary here) parameters that capture asymmetries penalize the IC model and make the CIMS model win by the BIC. Note also that the CIMS model is often rejected by the *G*^2^ statistic (stars in Figure 5) when it outperforms the IC model. Although statistical rejection is not indicative of a wrong model, this disagreement between *G*^2^ and the BIC emphasizes that the latter is more focused on economy than on the quality and interpretability of the fit (see García-Pérez & Alcalá-Quintana, 2012b). In fact because the BIC combines a measure of goodness of fit and a penalty based on the number of parameters used to achieve it, a model yielding a very poor fit may outperform a fitting model due to a larger penalty on the latter. This seems to be the case here with the IC and CIMS models.

### Effects of Stimulus Manipulations as Seen Through Model Parameters

**Figure 6.**
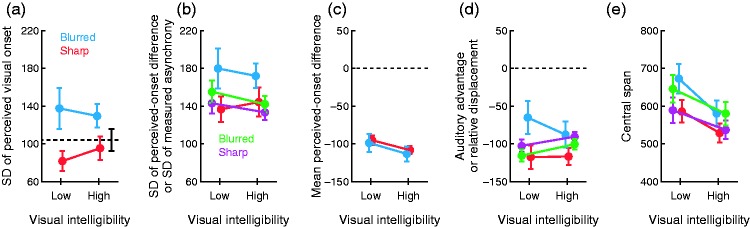
Parameter estimates across conditions. Estimates from the IC model are plotted with red and blue symbols, respectively, for conditions with sharp and blurred versions of the talking face image; corresponding estimates from the CIMS model (where applicable) are plotted with magenta and green symbols. Error bars are standard errors of the mean. (a) Standard deviations of perceived visual onsets. The dashed line indicates the standard deviation of perceived auditory onsets, assumed constant across conditions. (b) Standard deviations of perceived-onset differences (under the IC model) and measured asynchronies (under the CIMS model). (c) Means of perceived-onset differences. (d) Auditory advantage (under the IC model) or displacement (under the CIMS model). (e) Central span in decision space.

Estimated IC model parameters portray sensory and decisional aspects underlying observed differences across conditions. Consider the rate parameters λ_a_ (common across conditions) and λ_v_ (different for each condition), whose inverses are the standard deviations of the implied distributions of perceived onsets. The average σ_a_ = 1/λ_a_ across observers was 104.07 ms (see Table 2 in the Supplementary Information). On the other hand, the average σ_v_ = 1/λ_v_ was similar in conditions 1 and 2 (involving sharp images) and was also similar in conditions 3 and 4 (involving blurred images), with the latter pair being larger than the former. With rare exceptions, this pattern is apparent also at the individual level. Figure 6(a) shows these averages graphically. The dashed horizontal line is the average estimated standard deviation σ_a_ of perceived auditory onsets (invariant across conditions). To the naked eye, the average standard deviation σ_v_ of perceived visual onsets is smaller than the average σ_a_ when the image is sharp (red symbols lie below the dashed horizontal line in Figure 6(a)), but average σ_v_ exceeds average σ_a_ when the image is blurred (blue symbols lie above the dashed horizontal line in Figure 6(a)). A 2 × 2 repeated measures analysis of variance (ANOVA) with σ_v_ as the dependent variable revealed significant effects of image sharpness (*F*(1, 15) = 7.47; *p* = .015) but no effects of visual intelligibility (*F*(1, 15) < 1) and no interaction (*F*(1, 15) = 1.74; *p* = .207).

This specific analysis of the effects of stimulus manipulations is impossible under the CIMS model because it does not include separate parameters representing the distributions of perceived auditory and visual onsets. The CIMS model nevertheless estimates the variance of their difference (the variance σ^2^ of the distribution of measured asynchronies). The variance σ^2^ in the CIMS model has a counterpart in the variance $\sigma_{d}^{2}$ of the distribution of perceived-onset differences under the IC model. Figure 6(b) shows how estimates of σ (for the CIMS model) and σ*_d_* (for the IC model) vary across conditions. Although both models yield similar estimates, the CIMS model renders meaningfully smaller values with blurred images (green vs. blue symbols in Figure 6(b)). This is because the CIMS model cannot accommodate asymmetries in the data, which are generally larger with blurred images (see Figure 3). By fitting a symmetric model across data sets showing different degrees of asymmetry, standard deviations from the more asymmetric sets are underestimated. A 2 × 2 repeated measures ANOVA with σ*_d_* as the dependent variable revealed significant effects of image sharpness (*F*(1, 15) = 6.93; *p* = .019) but no effects of visual intelligibility (*F*(1, 15) < 1) and no interaction (*F*(1, 15) = 1.30; *p* = .272). These results contrast with those reported by Magnotti et al.(2013) in an analogous ANOVA with σ as the dependent variable: They reported a significant effect of visual intelligibility and a borderline effect of image sharpness, also with no interaction. This was replicated in our reanalysis.

A further aspect for which the CIMS model gives no output for comparison concerns the mean of the estimated distributions of perceived visual and auditory onsets when Δ*t* = 0 because the CIMS model assumes that the mean measured asynchrony is Δ*t* (see Figure 2(b)). Recall that these separate means cannot be estimated directly under the IC model (or any other model implying difference variables, for that matter) because their components τ_a_ and τ_v_ are combined into parameter τ. Nevertheless, the relative values of these means and how they vary across conditions can be assessed by looking at the mean perceived-onset difference when Δ*t* = 0, which is μ*_d_* = 1/λ_a_ − 1/λ_v_ + τ_a_ − τ_v_ (in contrast, this mean is assumed to be 0 under the CIMS model). As λ_a_ and τ_a_ are constant, any variations in this mean across conditions must reveal the effects of stimulus manipulations on mean perceived visual onset. The means μ*_d_* are plotted across conditions in Figure 6(c). A 2 × 2 repeated measures ANOVA revealed no significant main effects and no interaction.

The mean μ*_d_* of perceived-onset differences includes influences from the standard deviation of perceived visual onsets (i.e., 1/λ_v_) and the visual delay τ_v_ included in parameter τ, influences that may act in opposite directions to produce no effects on μ*_d_* (Figure 6(c)) despite significant effects on 1/λ_v_ (Figure 6(a)). To explore this possibility, we looked at how τ varied across conditions (Figure 6(d)). As discussed earlier, τ reflects the difference between the shortest possible auditory and visual perceived onsets when Δ*t* = 0, giving an indication of how manipulations of the visual component of the stimulus affect the shortest possible perceived visual onset under synchrony. Visual intelligibility of the spoken word did not affect auditory advantage with sharp images (red symbols in Figure 6(d)), but auditory advantage decreased meaningfully with blurred images (blue symbols in Figure 6(d)). This may seem counterintuitive but it suggests that blurred images make observers misjudge visual onset to occur arbitrarily earlier or later than they judge it to occur with sharp images, thus reducing auditory advantage (as seen in Figure 6(d)) and increasing the standard deviation of perceived visual onsets (as seen in Figure 6(a)). A 2 × 2 repeated measures ANOVA with τ as the dependent variable revealed significant effects of image sharpness (*F*(1, 15) = 8.26; *p* = .012) but no effects of intelligibility (*F*(1, 15) = 1.76; *p* = .204) and no interaction (*F*(1, 15) = 1.95; *p* = .183).

Parameter τ also reflects the location of the peak of the distribution of perceived-onset differences when Δ*t* = 0 and, thus, the shift of the distribution relative to the midpoint of the central region in decision space (see Figures 1(b) and 2(a)). This characteristic can be assimilated to an analogous aspect of the CIMS model that was discussed earlier, namely, that the CIMS model assumes instead a shift of the central region in decision space relative to the fixed position of the distribution of measured asynchronies (see Figure 2(b)). To assess how the IC and CIMS models compare as regards these displacements, Figure 6(d) also plots the relative displacement under the CIMS model, given by the negative value of the midpoint between δ^−^ and δ^+^. Estimated shifts are similar under both models, although they appear to change with image sharpness in opposite directions for each model.

A final aspect in which model accounts of the data can be compared concerns the width of the central region in decision space, that is, the central span given by 2δ in the IC model and by δ^+^ − δ^−^ in the CIMS model (see Figure 2). Figure 6(e) shows that the central span varies similarly across conditions under both models. A 2 × 2 repeated measures ANOVA with 2δ (for the IC model) as the dependent variable revealed significant effects of image sharpness (*F*(1, 15) = 7.56; *p* = .015) and also significant effects of visual intelligibility (*F*(1, 15) = 38.26; *p* < .001), with an interaction that did not reach significance (*F*(1, 15) = 4.29; *p* = .056). It should also be noted that the pattern of variation of the central span across conditions under the CIMS model (green and magenta symbols in Figure 6(e)) reproduces the pattern of variation of σ across conditions (green and magenta symbols in Figure 6(b)). This is a consequence of the structural property of the CIMS model by which δ^+ ^− δ^−^ and σ are monotonically related when μ_2_, σ_1_, σ_2_, and *p_C_*_ = 1_ have fixed values as they do here.

It should be remembered that the remaining parameters of the CIMS model (μ_2_, σ_1_, σ_2_, and *p_C_*_ = 1_) describe prior distributions and response biases assumed to be common across conditions and used to determine the boundaries δ^−^ and δ^+^ in combination with σ (see equation (5)). These parameters have no counterparts in the IC model, and no comparisons are possible beyond their effects on the central span (see Figure 6(e)). Further analyses of these parameters will not be conducted because they are uninterpretable due to the nonidentifiability discussed earlier.

### Conventional Performance Measures

**Figure 7.**
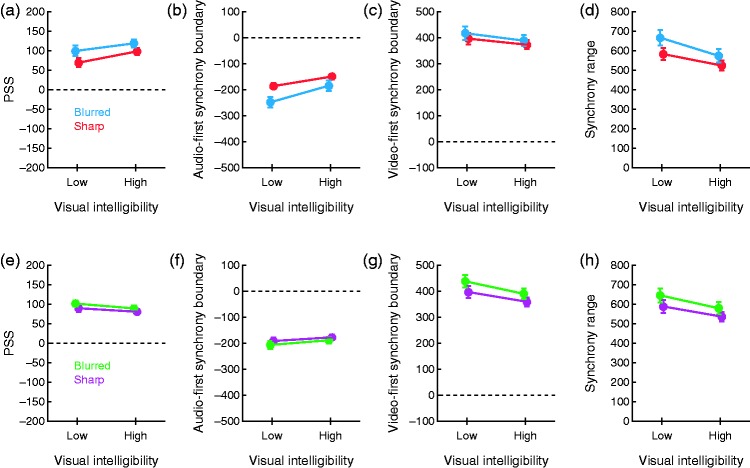
Performance measures across conditions. Measures obtained from the IC model are plotted with red and blue symbols, respectively, for conditions with sharp and blurred versions of the talking face image; corresponding estimates from the CIMS model are plotted with magenta and green symbols. Error bars are standard errors of the mean. (a) Average IC estimates of the PSS. (b) Average IC estimates of the audio-first synchrony boundary. (c) Average IC estimates of the video-first synchrony boundary. (d) Average IC estimates of the synchrony range. (e) Average CIMS estimates of the PSS. (f) Average CIMS estimates of the audio-first synchrony boundary. (g) Average CIMS estimates of the video-first synchrony boundary. (h) Average CIMS estimates of the synchrony range.

The PSS is defined as the audiovisual offset at which Ψ_S_ peaks. Under the IC model, the PSS is given by the expression for Δ*t*_peak_ given in section Models. When psychometric functions have a broad plateau (see Figure 3), peak location is hardly informative but Figure 7(a) shows how the average PSS varies across conditions. A 2 × 2 repeated measures ANOVA with Δ*t*_peak_ as the dependent variable revealed no effects of image sharpness (*F*(1, 15) = 3.12; *p* = .098), significant effects of visual intelligibility (*F*(1, 15) = 6.24; *p* = .025), and no interaction (*F*(1, 15) < 1). What the PSS tells is always unclear and these results attest to this fact: PSSs are immune to the effects of image sharpness and visual intelligibility that the preceding analyses disclosed.

We did not compute DLs because they are uninformative when Ψ_S_ has different drop-off rates on each side. As an alternative, we computed the AF and VF boundaries of the synchrony range, respectively defined as the left and right 50% points on Ψ_S_ (van Eijk et al., 2008). Computation of these boundaries under the IC model is described in Appendix A of García-Pérez and Alcalá-Quintana (2012b; see also Alcalá-Quintana & García-Pérez, 2013). Average AF and VF synchrony boundaries across conditions are plotted in Figure 7(b) and (c). To the naked eye, blurred images (blue symbols) push both synchrony boundaries further out compared with sharp images (red symbols), although the effect seems stronger on the AF synchrony boundary (Figure 7(b)). This reflects the increased asymmetry of the psychometric functions caused by distributions of perceived visual onsets that have a larger standard deviation with blurred images: Compared with sharp images (red symbols), PSSs are higher with blurred images (blue symbols in Figure 7(a)) but AF synchrony boundaries are lower (blue symbols in Figure 7(b)), whereas VF synchrony boundaries remain virtually identical (blue symbols in Figure 7(c)). Separate 2 × 2 repeated measures ANOVAs were conducted with each synchrony boundary as the dependent variable. For the AF boundary, the analysis detected significant effects of image sharpness (*F*(1, 15) = 9.72; *p* = .007), significant effects of visual intelligibility (*F*(1, 15) = 74.75; *p* < .001), and no interaction (*F*(1, 15) = 2.65; *p* = .124); for the VF boundary, the analysis did not detect effects of image sharpness (*F*(1, 15) = 1.94; *p* = .184) but detected marginally significant effects of visual intelligibility (*F*(1, 15) = 5.04; *p* = .040) also with no interaction (*F*(1, 15) < 1). Figure 7(d) shows the average synchrony range (the difference between the VF and the AF synchrony boundaries), reflecting the range of audio or visual offsets within which judgments of synchrony prevail. As expected from results in Figure 7(b) and (c), the synchrony range is broader with blurred images, indicating less ability to perceive asynchronous speech. A 2 × 2 repeated measures ANOVA with synchrony range as the dependent variable revealed significant effects of image sharpness (*F*(1, 15) = 7.23; *p* = .017), significant effects of visual intelligibility (*F*(1, 15) = 39.66; *p* < .001), and no interaction (*F*(1, 15) = 4.11; *p* = .061).

We also computed analogous performance measures from the symmetric psychometric functions provided by the CIMS model, and the results are plotted in Figure 7(e) to (h). Symmetric psychometric functions wash out differences in asymmetry across conditions and render PSSs that are much more similar (Figure 7(e)), synchrony boundaries that are also much more similar (Figure 7(f) and (g)) and synchrony ranges that only reflect overall differences (Figure 7(h)). Note that the AF and VF synchrony boundaries are necessarily symmetrically placed with respect to the PSS when they are extracted from symmetric functions. Thus, differences in AF versus VF synchrony boundaries with blurred or sharp images (compare the distance between green and magenta lines in Figure 7(f) and (g)) only reflect the differences in PSSs shown in Figure 7(e) and the differences in breadth shown in Figure 7(h): In these conditions, the AF synchrony boundaries with blurred versus sharp images must be closer to one another (Figure 7(f)) and the VF synchrony boundaries with blurred versus sharp images must accordingly be farther from one another (Figure 7(g)). This artifact of enforced symmetry does not reflect independent effects of stimulus conditions on synchrony boundaries.

## Discussion

Our analyses of Magnotti et al.’s (2013) data under the IC model have shown that manipulation of the visual component of audiovisual speech stimuli affects observed performance in a way that can be expressed in terms of the distribution of perceived visual onsets. The effects of these manipulations on the asymmetry of the data were also adequately captured by the model. Our comparison of the IC and CIMS models has also shown that the CIMS model cannot capture these effects due to its symmetric psychometric functions and the absence of explicit representations of perceived auditory and visual onsets. The IC model fitted asymmetric data better than the CIMS model even as measured by the BIC; for symmetric data, the CIMS model outperformed the IC model by the BIC (due to the penalty based on the number of parameters) and despite the fact that the CIMS model was indeed rejected in most of those cases by the goodness-of-fit test. Asymmetry is a widespread characteristic of SJ2 data (see Keetels & Vroomen, 2012) and it is impossible to know beforehand whether some observers (or some conditions) will render data that will adequately be accounted for by a model that assumes symmetry. The following sections discuss other issues prompted by our analyses.

### Methodological and Experimental Approaches in Studies on Synchrony Perception

Fitting model-based psychometric functions with interpretable parameters to timing judgment data has numerous advantages over using other types of function (García-Pérez & Alcalá-Quintana, 2012a, 2012b, 2015a, 2015b; Matsuzaki et al., 2014; Regener et al., 2014). The most apparent advantage is that typical data features such as asymmetries and plateaus can be properly captured, but the most distinctive advantage is that interpretable parameters offer unparalleled insights into the processes underlying timing judgments, also offering the means for testing hypotheses about such processes or the stimulus- and task-dependent influences affecting them.

Our analyses exemplify how such effects can be assessed across within-subjects conditions involving degradations of the visual signal. One can envision an analogous use of models for assessing the effects of degradations of the auditory signal (where the model would be fitted by keeping the visual component invariant) or in designs in which the visual and auditory components are both manipulated and crossed: Models would be fitted by keeping the visual (or auditory) component invariant across conditions involving the same visual (or auditory) manipulation.

### Response Errors and Efficient Data Collection Strategies

We have used the IC model without its extension to account for response errors. Such extension adds extra parameters and its use should be guided by evidence of response errors, which shows at large positive and negative audiovisual offsets where synchronous judgments should never be reported. Thus, the occurrence of synchronous responses in this region is a dependable indicator of response errors (García-Pérez & Alcalá-Quintana, 2012a, 2012b, 2015a, 2015b). Except perhaps for observers #7 and #11 (see Figure 3), the data analyzed here did not cover the full breadth of the psychometric function so that evidence of response errors was not patent and the use of the extended model was unwarranted.

The data had been collected with the method of constant stimuli (MOCS). In general, MOCS does not ensure adequate sampling of the psychometric function: Sometimes the predefined range of audiovisual offsets turns out to be too broad; other times, it is too narrow or misplaced. These eventualities also affect the possibility of obtaining evidence of response errors and they are difficult to anticipate when the effects of experimental manipulations are unknown, particularly on consideration of large individual differences. Adaptive methods overcome these difficulties by placing trials where it seems relevant given each observer’s performance. Adaptive methods for use with nonmonotonic psychometric functions have been developed and proven superior to MOCS (García-Pérez, 2014). The use of efficient data collection strategies will also result in more dependable and informative data for assessing the effects of experimental manipulations and for investigating differences between groups (e.g., patients and normal controls).

## Conclusion

Research on perception of asynchronous speech or, generally, on perception of temporal order has theoretical and practical ramifications. Studies generally collect data with MOCS and analyze them by fitting arbitrary psychometric functions. Identifiable models of timing judgments offer alternative psychometric functions with interpretable parameters that allow looking into the processes that determine observed performance, and adaptive methods offer optimal strategies for the collection of maximally informative data. Use of within-subjects designs, adaptive methods, and model-based psychometric functions fitted jointly across conditions under the applicable constraints can only result in more efficient and conclusive research, supporting Greenwald’s (2012) claim that there is nothing so theoretical as a good method.

## Supplementary Material

Supplementary material
