# Supplementary material for: Visual and Auditory Components in the Perception of Asynchronous Audiovisual Speech
Source: Iperception. 2015 Nov 30;6(6):2041669515615735. doi: 10.1177/2041669515615735 (PMC4975115; doi:10.1177/2041669515615735)
Supplement: Supplementary material [file Supplementary_Material_735.pdf]

Visual and auditory components in the perception of asynchronous audiovisual speech

Miguel A. García-Pérez and Rocío Alcalá-Quintana
Departamento de Metodología, Facultad de Psicología, Universidad Complutense,
Campus de Somosaguas, 28223 Madrid, Spain

Supplementary Information

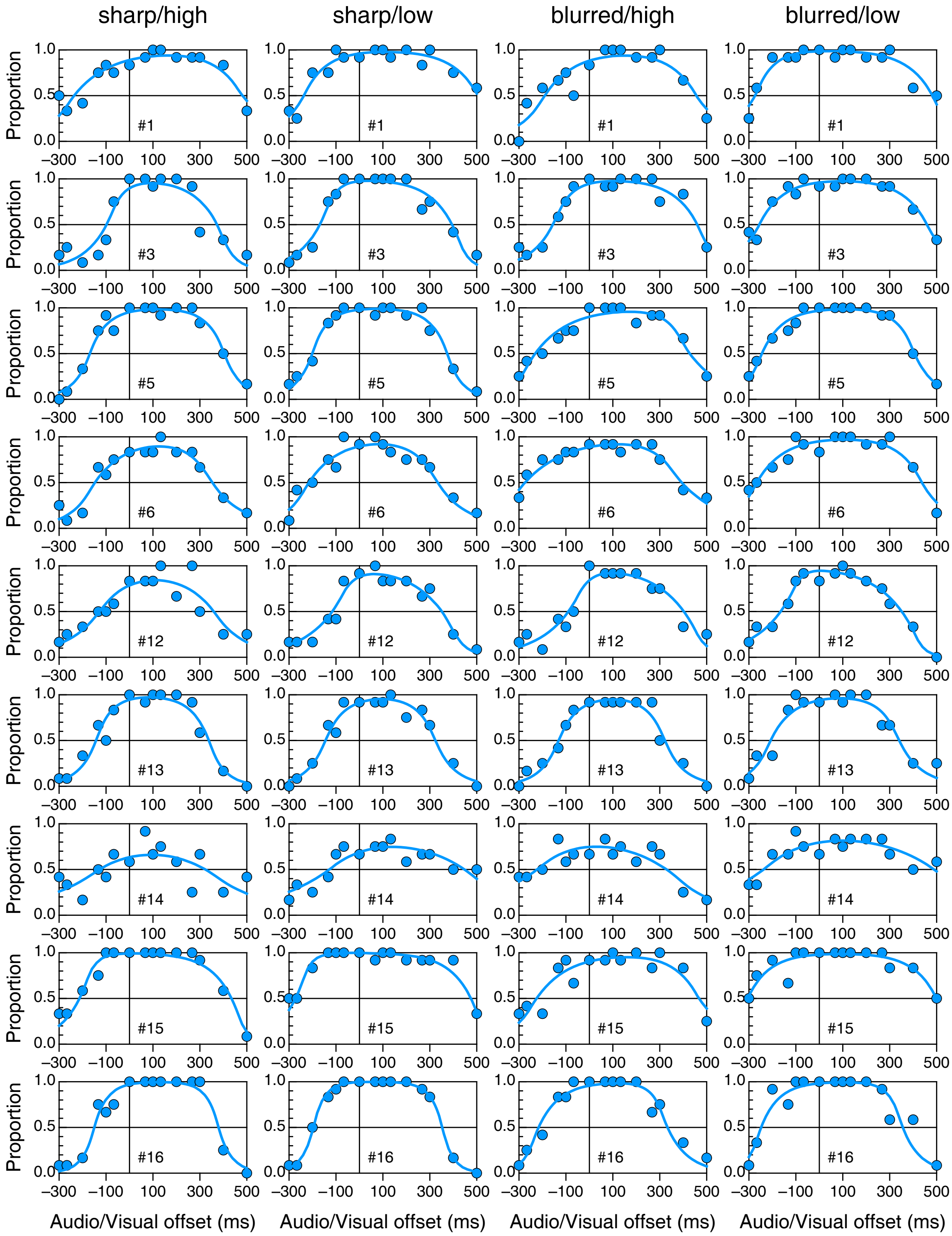

Supplementary Figure S1. Data and fitted psychometric functions from the IC model for observers not shown in Figure 3. Layout as in Figure 3.

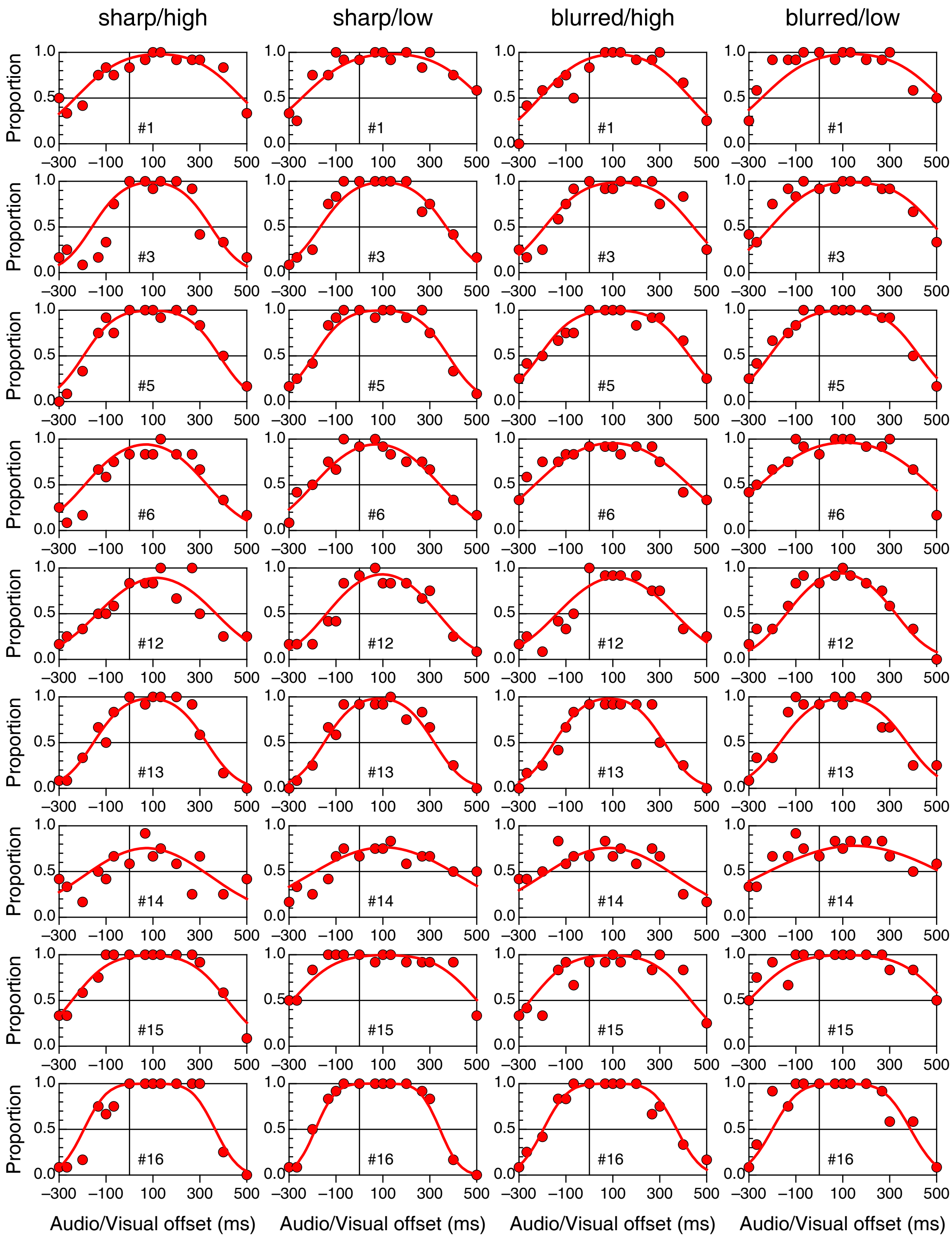

Supplementary Figure S2. Data and fitted psychometric functions from the CIMS model for observes not shown in Figure 4. Layout as in Figure 4.

Supplementary Table 1. List of IC and CIMS model parameters

IC model parameters	
λ_a	Rate parameter of the distribution of perceived auditory onsets
λ_v	Rate parameter of the distribution of perceived visual onsets
τ_a	Processing delay in the distribution of perceived auditory onsets
τ_v	Processing delay in the distribution of perceived visual onsets
δ	Temporal resolution limit
	Auditory advantage parameter ($\tau = \tau_a - \tau_v$)

CIMS model parameters	
σ	Standard deviation of the distribution of measured asynchronies
μ_1	Mean of the prior distribution of asynchronies under a single cause ($\mu_1 = 0$ by assumption)
μ_2	Mean of the prior distribution of asynchronies under two causes
σ_1	Standard deviation of the prior distribution of asynchronies under a single cause
σ_2	Standard deviation of the prior distribution of asynchronies under two causes
$p_{C=1}$	Observer's bias towards assuming a single cause

Supplementary Table 2. IC model parameter estimates

Estimated parameters under the IC model for each observer. The columns labeled G^2 and p respectively give the value and p-value of the likelihood-ratio goodness-of-fit statistic with 47 degrees of freedom; the star for observer 11 indicates rejection at the 5% significance level. The bottom rows give the mean and standard deviation (SD) of each parameter across observers.

Obs.	G^2	p	$1/\lambda_a$	$1/\lambda_v^{(1)}$	$1/\lambda_v^{(2)}$	$1/\lambda_v^{(3)}$	$1/\lambda_v^{(4)}$	$\tau^{(1)}$	$\tau^{(2)}$	$\tau^{(3)}$	$\tau^{(4)}$	$\delta^{(1)}$	$\delta^{(2)}$	$\delta^{(3)}$	$\delta^{(4)}$
1	56.61	0.159	104.95	151.51	89.43	127.43	67.50	−98.43	−142.04	−120.95	−124.70	359.37	370.92	323.66	377.86
2	54.64	0.207	124.63	72.17	52.44	119.65	109.45	−152.32	−135.83	−93.54	−126.33	310.35	376.42	401.09	429.14
3	57.62	0.138	99.91	53.82	46.01	65.17	95.26	−163.37	−144.29	−171.10	−103.96	235.90	281.85	307.54	357.10
4	58.61	0.119	59.97	64.90	114.36	193.48	234.21	−97.45	−26.26	−39.95	22.11	163.48	196.45	296.73	320.03
5	38.70	0.800	68.41	70.51	59.54	140.73	85.62	−116.27	−91.42	−57.48	−71.42	286.46	287.72	330.19	325.25
6	36.10	0.876	91.93	130.99	125.96	175.32	110.32	−82.40	−40.94	−13.88	−75.77	251.57	274.84	336.01	351.71
7	45.19	0.548	75.79	59.47	88.31	135.12	141.94	−104.37	−72.83	−63.14	3.21	202.11	232.54	174.41	197.02
8	49.48	0.375	101.45	112.47	46.00	79.16	105.09	−132.83	−176.45	−181.54	−126.45	290.79	325.08	233.39	317.84
9	44.87	0.561	100.23	124.73	103.98	242.11	406.20	−88.93	−132.74	48.56	180.38	308.96	350.76	383.60	516.84
10	40.30	0.744	136.72	35.00	39.63	106.24	228.52	−230.02	−248.34	−177.81	−142.81	295.92	363.97	323.14	372.36
11	64.69 *	0.044	86.55	146.39	143.27	168.18	135.55	−9.92	−8.19	−4.77	30.59	192.08	203.43	146.49	201.83
12	42.71	0.651	140.18	126.49	55.79	55.78	38.73	−122.02	−165.08	−204.60	−154.38	246.92	235.78	248.24	259.98
13	47.66	0.446	73.24	61.61	79.54	78.51	93.82	−104.07	−85.80	−92.27	−55.24	240.41	234.81	227.05	273.97
14	40.31	0.744	258.38	223.09	187.46	165.36	183.46	−111.73	−190.58	−94.49	−161.29	260.22	306.20	293.62	373.03
15	50.17	0.349	95.53	40.42	36.01	133.01	91.36	−143.16	−136.96	−101.26	−99.03	324.08	373.90	346.51	402.79
16	46.02	0.513	47.17	53.94	42.31	88.06	75.09	−110.83	−81.12	−35.21	−38.38	264.87	274.79	274.80	301.83
Mean			104.07	95.47	81.88	129.58	137.63	−116.76	−117.43	−87.71	−65.22	264.59	293.09	290.40	336.16
SD			47.02	50.05	42.26	49.30	87.18	44.51	62.05	69.09	86.47	50.02	60.01	67.88	78.97
